# A Review of the Uncertainties of Eukaryotic Translation Initiation

**DOI:** 10.3390/genes17070800

**Published:** 2026-07-13

**Authors:** Anton A. Komar, William C. Merrick

**Affiliations:** 1 Center for Gene Regulation in Health and Disease, Department of Biological, Geological and Environmental Sciences, Cleveland State University, Cleveland, OH 44115, USA; a.komar@csuohio.edu; 2 Department of Biochemistry, School of Medicine, Case Western Reserve University, Cleveland, OH 44106, USA

**Keywords:** mRNA, ribosome, initiation factor, eIF4A, eIF4F disassembly, scanning, ATP, initiation of eukaryotic protein synthesis, equilibrium in assembly

## Abstract

This article proposes three possible explanations that relate to the process of eukaryotic translation initiation. These explanations suggest mechanisms as to how concentrations of initiation factors (by mass or by posttranslational modification) can influence start site selection, how regulation of translation by 4E-BP (an inhibitor of m^7^G cap-dependent translation) can be explained by “eIF4F disassembly” and how the scanning mechanism might involve initiation factor binding at the 5′ end of the mRNA to provide for apparent unidirectional Brownian movement to locate the initiating AUG. These represent testable models, although the experiments would not be simple. It is hoped that the insights provided will assist researchers in defining the precise steps and mechanisms of the complex initiation process. It is noted that the better this process is understood, the easier it will be to understand how this process is regulated under a wide variety of biological situations, such as nutritional deprivation, heat shock, cell growth, or disease. Additionally, as initiation is the rate-limiting step in translation, a better understanding of this process should also suggest new avenues to treat various diseases, especially conditions of unrestricted growth.

## 1. Introduction

Over the past decades, an enormous amount of information has been acquired that relates to the mechanism and regulation of eukaryotic protein synthesis initiation. This article intends to provide possible mechanisms for current problems under study.

**Figure 1:**
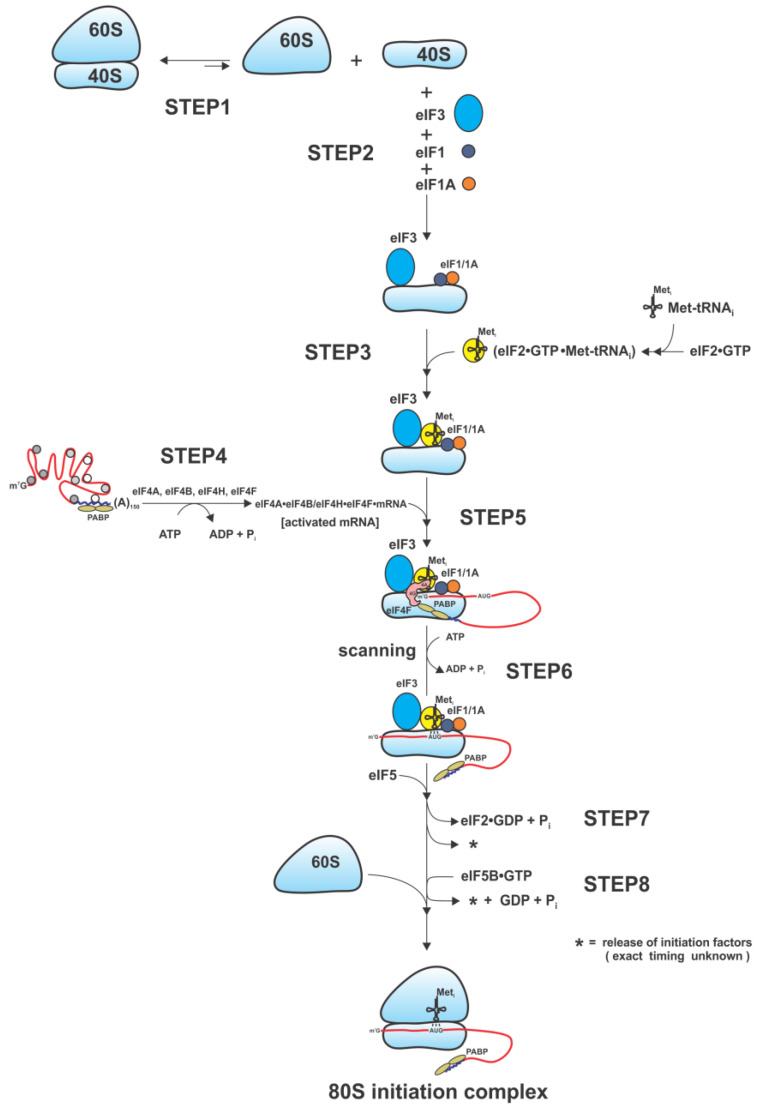
The standard pathway for m^7^G cap-dependent translation initiation. While all of the canonical initiation factors are indicated, the precise timing for their addition to the 40S subunit or their subsequent departure is not known. The reader is encouraged to see one of the recent review articles for greater detail [1,2,3,4,5]. Note: For those less familiar with the eukaryotic translation initiation factors, a brief description of each is provided in Appendix A.

While a wide variety of alternate mechanisms keep evolving, the primary initiation mechanism in growing cells is the m^7^G cap-dependent pathway. A representation of this pathway is shown in Figure 1. The basic steps appear to be as follows:

1. Dissociation of 80S ribosomes into the 40S and 60S subunits.
2. Binding of eIF1, eIF1A, and eIF3 to the 40S subunit (which prevents rebinding to the 60S subunit).
3. Binding of the ternary complex of eIF2•GTP•met-tRNA_i_ to the 40S•eIF1, -1A, -3 complex (which will facilitate identifying the start codon).
4. ATP-dependent activation and then subsequent binding of the mRNA by eIF4A, eIF4B, and eIF4F.
5. Binding of the activated mRNA (with the eIF4 proteins) to the 40S subunit.
6. Scanning of the mRNA in a 5′ to 3′ direction until the AUG start codon is reached.
7. With the matching of the met-tRNA_i_ to the initiating AUG codon, hydrolysis of the GTP and release of eIF2•GDP + Pi.
8. Binding of eIF5B•GTP to the 40S complex (with mRNA and met-tRNA_i_) and joining of the 60S subunit, which triggers the hydrolysis of the GTP and release of eIF5B.

While these are the generally agreed-upon steps, how they are accomplished is less certain. The reader is encouraged to view a number of reviews on this topic for a more complete understanding of this process and, importantly, its regulation [1,2,3,4,5]. Some of the remaining questions are as follows: What is the exact timing for the initial binding and release of the initiation factors and the mechanism of scanning? Is this initiation pathway truly unidirectional, or may there be several “equilibrium” steps in the process? The description below provides a possible explanation for each of these questions.

## 2. Possible Explanation 1—There Are Equilibrium Steps in the Initiation Process

**Figure 2:**
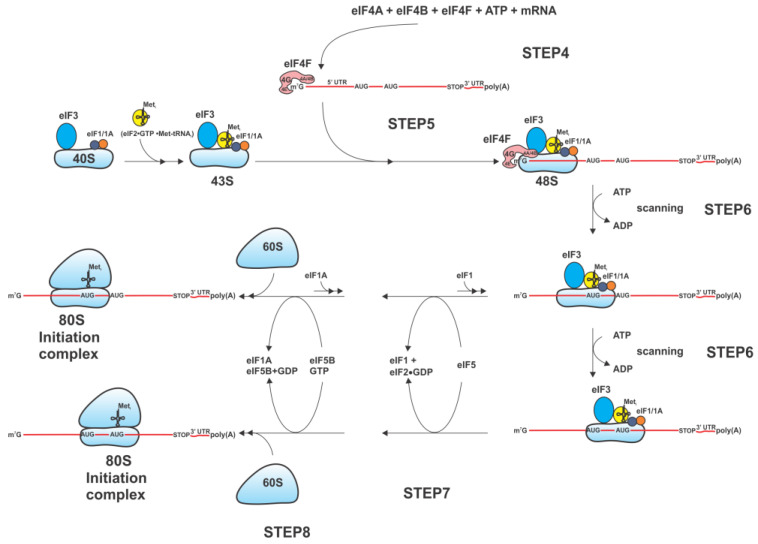
The postulation of equilibrium (or quasi-equilibrium) steps in the process of eukaryotic translation initiation. The flow diagram depicts the possible equilibrium steps involved with eIF1, eIF1A, eIF5, and eIF5B. In this scheme, excess eIF1 or eIF1A allows for rebinding and thus enhances the accuracy of the translation initiation. Where a non-Kozak consensus sequence is encountered at the 5′ most possible start site, a rebinding of either of these proteins favors continued scanning. In the presence of excess eIF5 or eIF5B, the utilization of the 5′ most start codon is favored even if it is not a good Kozak consensus sequence.

From Figure 1, there is already an equilibrium process in that there is an equilibrium between 80S ribosomes and their two subunits (40S and 60S). However, the rest of the pathway would appear to be unidirectional. A number of studies have examined the effects of altering one or more of the following proteins: eIF1, eIF1A, eIF5, eIF5B, or protein pairs (eIF1 and eIF5 or eIF1A and eIF5B) [6,7]. However, most of these studies assessed the influence of these proteins using a single reporter, which may or may not have contained alterations in the test mRNA. To assess if an increase in any of the canonical initiation factors might influence start site selection, we sampled six different mRNA constructs, each with two or more possible start codons (either AUG or CUG) [8]. What we found was that when the concentrations of eIF1 or eIF1A were elevated, the preferred start codon selected was based upon a perfect match with the Kozak consensus sequence [9]. When either eIF5 or eIF5B was elevated, then the start codon nearest the 5′ end of the mRNA was selected. We note that eIF1 serves as the primary gatekeeper, enforcing stringent recognition of the correct start codon, whereas eIF5 functions as a GTPase-activating protein that promotes eIF1 dissociation, thereby committing the ribosome to translation initiation [6,7]. These observations led us to propose the model shown in Figure 2.

In this model, with the release of eIF1, when eIF1 (or eIF1A) is in excess, it is possible to rebind eIF1 and then continue scanning to select the second AUG start codon. In contrast, excess eIF5 or eIF5B pulls the ribosome complex towards subunit joining after the first start codon has been reached. Thus, the accuracy of start codon selection will be a balance between the concentrations of eIF1, eIF1A, eIF5, and eIF5B. This conclusion is consistent with the reports from others, using alternate methods of analysis [10,11,12].

We also note that one of the notable features of eukaryotic translation initiation is the autoregulatory control of several core initiation factors [13,14,15]. Curiously, of the three initiation factors under autoregulation control, two are eIF1 and eIF5 [13,14], and the third is eIF4G [15]. Autoregulation implies that when the levels of a specific protein are high, in some manner, it causes a downregulation of its own expression. Conversely, when concentrations of the protein are low, an upregulation of expression is triggered. In the case of eIF1, the initiating AUG is in a poor Kozak context, and thus initiation from this start site is poor when eIF1 levels are high (and drives enhanced accuracy for the start codon) [13]. When the concentration of eIF1 is low, there is poorer start codon selectivity and thus, expression of eIF1 is upregulated. In the case of eIF5, when its levels are high, upstream uORFs are recognized, which leads to a decreased expression of eIF5 [14]. Conversely, when eIF5 concentrations are low, there is enhanced selectivity of the authentic AUG start codon; thus, there is an elevation of eIF5 expression.

The case for eIF4G is a bit more complex. To begin with, m^7^G cap-dependent initiation is much more efficient than any of the alternate pathways for initiation (internal ribosome entry site (IRES)-mediated, cap-independent, re-initiation, etc.). Thus, when eIF4G (and consequently eIF4F) levels are high, m^7^G cap-dependent initiation is favored and outcompetes the other mechanisms. However, when levels of eIF4G are low (and subsequently, also eIF4F), the alternate pathways have an opportunity for expression. The 5′ UTR of eIF4G contains an IRES [15]; thus, its expression is optimal under conditions where eIF4F-dependent translation is markedly reduced. A reduction in eIF4F activity could be the result of low concentrations of eIF4G or reduced access of eIF4G to eIF4E, which could be tied up in an eIF4E•4E-BP complex. The ability of 4E-BP to bind eIF4E is controlled by phosphorylation via mTOR, with the phosphorylated form being inactive in eIF4E binding.

It should be noted from Figure 2 that this does not address other possible equilibrium steps which could be associated with eIF3 binding to 40S subunits, binding of the ternary complex to 40S subunits, the scanning process (is it only 5′ to 3′?), the binding of eIF5B•GTP to 40S subunits, or subunit joining.

## 3. Possible Explanation 2—eIF4F Disassembly

One of the steps in regulating cap-dependent protein synthesis initiation is the regulation by 4E-BP [1,2,3,4,5]. As its name suggests, it can bind to eIF4E, and when it does, it blocks the ability of eIF4G to bind eIF4E to form the eIF4F complex, as 4E-BP and eIF4G bind to overlapping sites on eIF4E such that eIF4E can be bound to one of the proteins or the other, but not both. However, the original observation based upon a six-day purification scheme for eIF4F suggests that the complex is quite stable [16]. If this is true, how would 4E-BP ever have a chance to bind eIF4E? Several experimental results have indicated that while eIF4F is stable in isolation, other components can labilize the complex and cause one or both of the subunits to separate from the scaffolding eIF4G subunit.

The initial observations of eIF4F instability reflected our efforts to improve the purity of particular preparations of eIF4F. By subjecting a slightly impure preparation of eIF4F to chromatography on phosphocellulose, the complex of eIF4E•eIF4G was retained on the column while the eIF4A subunit was released from the column [17]. It was inferred that the phosphocellulose matrix was mimicking RNA and that this triggered the release of the eIF4A subunit. In an alternate purification scheme, partially purified eIF4F was bound to an m^7^G affinity column. To get rid of impurities, the column was washed extensively with buffer. In the end, when the eIF4F was to be eluted with m^7^GDP, all that was recovered was eIF4E (Merrick, unpublished observation; see also ref. [18,19]). Thus, the binding of eIF4E to the cap structure labialized the eIF4E association with the eIF4A•eIF4G complex.

**Figure 3:**
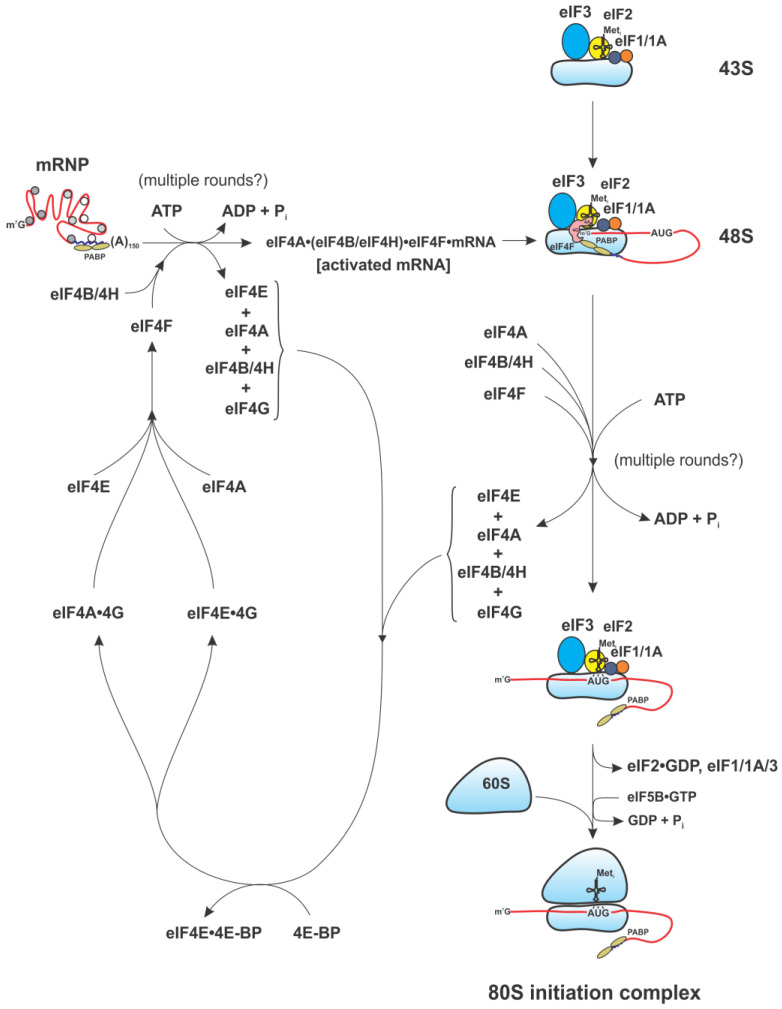
Possible eIF4F disassembly. A diagram of the two possible sites where eIF4F may undergo disassembly is shown. Also shown is the possible binding of eIF4E to 4E-BP to form the inactive eIF4E•4E-BP complex.

It is assumed that both of these observations in some manner lead to the separation of the eIF4F subunits during the process of initiation. Additional evidence of this separation was observed when eIF4A, eIF4B, and eIF4F were incubated with oxidized radiolabeled reovirus RNA (ref. [20], Figure 3). In the absence of ATP, only eIF4E was labeled. In the presence of ATP, eIF4A and eIF4B were labeled. In the presence of ATP and m^7^GDP, there was a reduction in the labeling of eIF4A and eIF4B. The loss of labeling of eIF4E in the presence of ATP and the oxidized reovirus RNA suggests that the association of eIF4E with eIF4G is labialized by the binding of the m^7^G cap to eIF4E (similar to the results with the m^7^G affinity column).

The result is that eIF4F appears to disassemble during the process of initiation. However, it is not possible to tell how much of this might occur during the mRNA activation step and how much on the surface of the 40S subunit as might be associated with scanning (Figure 3). The key feature here is that with each round of initiation, the eIF4E subunit becomes free and thereby becomes accessible to regulation via 4E-BP, whereas the stable eIF4F complex would never present a binding site in its eIF4E subunit to 4E-BP. The suggestion that eIF4E might dissociate from the eIF4F complex has been made previously [21].

The activity of 4E-BP is regulated primarily through phosphorylation by the serine/threonine kinase mTOR, specifically the mTOR complex 1 (mTORC1) [22,23]. Under nutrient-rich and growth-promoting conditions, mTORC1 phosphorylates 4E-BP at multiple sites, reducing its affinity for eIF4E and thereby permitting assembly of the eIF4F complex to promote cap-dependent translation initiation. Conversely, the inhibition of mTORC1 results in hypophosphorylated 4E-BP, which tightly binds to eIF4E, preventing its association with eIF4G and suppressing cap-dependent translation. As such, with the release of eIF4E from the eIF4F complex during each round of initiation, eIF4E becomes available for binding by and regulation through 4E-BP [22,23]. Through this mechanism, the mTORC1–4E-BP signaling axis serves as a central regulator that links cellular nutrient availability, energy status, growth factor signaling, and stress responses to the control of protein synthesis [22,23].

Recent studies have also provided new insights into how heat stress regulates translation initiation. Heat shock rapidly inhibits cap-dependent translation by disrupting early initiation events, including displacement of scanning initiation factors such as eIF4A from translating ribosomes [24]. In parallel, heat stress induces the formation of “silent” ribosomes that are devoid of mRNA, suggesting that mRNA recruitment to the small ribosomal subunit is impaired [25]. Together, these findings highlight the sensitivity of the earliest steps of translation initiation to cellular stress and underscore their importance in translational reprogramming.

Undoubtedly, cellular stress plays an important role in regulating translation initiation through multiple mechanisms that modulate initiation factor availability, ribosome engagement, and mRNA recruitment [1,2,3,4,5]. However, a detailed discussion of these stress-responsive pathways is beyond the scope of this short opinion/review article.

## 4. Possible Explanation 3—The Role of eIF4A in Scanning

As characterized by Cryo-EM, the eIF4 proteins appear to be bound to 40S subunits at the exit site of the ribosome [26,27,28,29]. This makes sense in that this is where the 5′ end of the mRNA is, and the 5′ of the decoding site is important for the recognition of the initiating AUG codon. This pictures the mRNA on the 40S subunit, but not after scanning to the initiating AUG. As this appeared to be an ATP-dependent process [30], the key player thought to be associated with scanning was eIF4A, the only initiation factor that binds ATP. However, enzymatically, it was difficult to determine how this might happen.

The problem is that the RNA helicases to date have rather low turnover numbers, on the order of 2 or 3 reactions per minute (see Table 1 and ref. [31,32,33,34]), including the yet uncharacterized role of a second RNA helicase, Ded1 (in humans, DDX3).

Secondly, studies on model RNA duplex substrates indicated that the DEAD-box RNA helicases bound to the double-stranded region and directly pried apart the duplex rather than using the DNA helicase-type activity of binding to a single strand and then translocating through the duplex region to unwind it. Thus, with no directional motor to drive scanning, it had been suggested by several research groups that Brownian movement might account for the scanning process [35,36]. The problem here is that Brownian movement is not directional. So how could this be modified to impart a direction?

**Figure 4:**
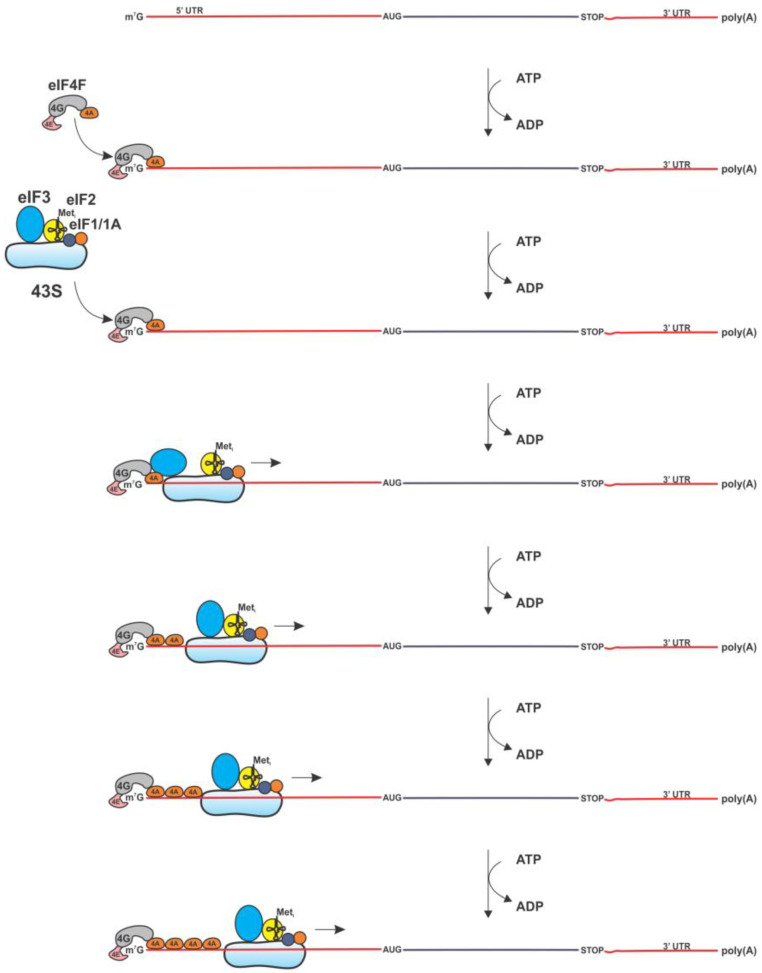
A possible mechanism to ensure unidirectional scanning in the 3′ direction. Following the initial binding of eIF4F and the subsequent binding of the eIF4F•mRNA complex to the 43S preinitiation complex, Brownian movement (sometimes referred to as linear diffusion) allows for movement of the mRNA on the 40S subunit. Successive rounds of eIF4A binding block movement in the 5′ direction. These eIF4A molecules could be derived from free eIF4A or free eIF4A cycled through the 40S-bound eIF4G or from additional molecules of eIF4F.

One idea is that proteins might bind on the 5′ side of the codon recognition site and thereby prevent movement in the 5′ direction. Such an example is shown in Figure 4. In this case, the blocking protein is eIF4A. There are two reasons why this might make sense. The first is that this would offer an explanation for why scanning might be an ATP-dependent process. The slow turnover number for eIF4A as an RNA helicase actually would suggest that it is more of a clamp, as previous studies had shown that the off rate of eIF4A from bound RNA was on the order of 30 s [33]. Secondly, the use of multiple eIF4A molecules would be consistent with the observation of studies with a dominant negative eIF4A mutant, which concluded that there must be multiple rounds of eIF4A usage (or eIF4F usage, see ref. [37]. Third, it had also been previously shown that free eIF4A can exchange for the eIF4A bound in the eIF4F complex [38] and that there were two binding sites for eIF4A on eIF4G, although when purified, the eIF4F complexes appeared to have only a single eIF4A contained in it [16]. What is not clear in this model is whether the additional eIF4A molecules are recruited from free eIF4A (which is 5 to 10 times more abundant than the other initiation factors) or whether it is cycled through an eIF4G•eIF4E complex or perhaps just eIF4G bound at the 5′ exit site. It should be noted that the concept of unidirectional scanning from 5′ to 3′ has been made, with some documentation, previously [39,40].

If eIF4A facilitates a net 5′ to 3′ linear diffusion, how does it stop? One key is obviously the initiator tRNA bound in the ternary complex, which is assumed to sample all possible codons during scanning. An alternate that has been shown in a model system by Marilyn Kozak, secondary structure could slow down or stop scanning and facilitate initiation at a non-ideal start codon (either poor consensus or non-AUG) [41]. Given the large number of nucleotides, it is highly likely that many protein-coding regions have extensive secondary structure and could easily be a brake on any further movement in the 3′ direction. As an example, the structure map for mouse beta globin mRNA is almost perfectly double-stranded, such that one could do hyperchromicity studies with this mRNA [42].

## 5. Conclusions

The primary purpose of proposing the above possible explanations is to stimulate experimentation to explore the unknown. It is hoped that these will guide or inspire research efforts that will ultimately lead to concrete answers as they relate to the very complicated process of protein biosynthesis initiation, at least for the m^7^G cap-dependent process.

The wide variety of cellular responses to stress or disease leaves open a mysterious array of other events that will be even more challenging to fully decipher.

## Figures and Tables

**Table 1:** Enzymology of the DEAD-box RNA helicases.

The Average Turnover Number is Roughly 2 to 3 Reactions per Minute (Supporting Evidence From the Literature is Listed Below) ^1^		
Literature Source	Supporting Evidence	Ref.
Lorsch and Herschlag, 1998	eIF4A (RNA-dependent ATPase) activity	[31]
Sharma et al., 2017	Ded1 unwinding	[32]
Iwasaki et al., 2016	eIF4A off rate from RNA	[33]
Andreou et al., 2017	RNA-dependent ATPase with eIF4A or eIF4A + eIF4B + eIF4G	[34]

^1^ For each of the cited publications, the rate of ATP hydrolysis or the off rate for a protein•RNA complex was determined to be less than three reactions per minute (1, 2, and 4) or an off rate from the protein•RNA complex of more than 30 s (3).

## Data Availability

No new data were created or analyzed in this study. Data sharing is not applicable to this article.

## Abbreviations

The following abbreviations are used in this manuscript:

eIF	Eukaryotic Initiation Factor
Cryo-EM	Cryogenic Electron Microscopy

## Appendix A

**Table A1:** The key protein factors required for translation initiation. For those who are not steeped in the intricacies of the “factor” world of translation initiation, presented below is a list of the proteins involved and a simple explanation of their activity in the translation initiation process.

Protein	Molecular Weight (Da)	Function
eIF1	12,500	ensures the accuracy of start codon identification
eIF1A	16,500	ensures the accuracy of start codon identification
eIF2 *	125,000	forms a ternary complex with GTP and Met-tRNAi
eIF2B *	260,000	recycles eIF2•GDP complexes
eIF3 *	650,000	builds pool of free 40S subunits, provides the protein contact for the eIF4F•eIF4A•eIF4B/4H•mRNA complex to bind to 40S subunits
eIF4A	45,000	ATP-dependent RNA helicase
eIF4B *	140,000	stimulates the activities of eIF4A and eIF4F
eIF4E	25,000	recognizes the m^7^G cap of mRNAs
eIF4G	170,000	scaffolding protein that provides contacts for eIF4E, eIF4A, and eIF4B
eIF4F *	240,000 (4A, 4E, 4G)	binds m^7^G cap of mRNAs and unwinds secondary structures in the 5′ UTR
eIF4H	25,000	stimulates the activities of eIF4A and eIF4F
eIF5	49,000	triggers GTP hydrolysis in the eIF2 ternary complex
eIF5B	125,000	drives GTP-dependent subunit joining

* = multisubunit proteins.
